# MiR-136 modulates glioma cell sensitivity to temozolomide by targeting astrocyte elevated gene-1

**DOI:** 10.1186/s13000-014-0173-0

**Published:** 2014-09-30

**Authors:** Hao Wu, Qiang Liu, Tao Cai, Yu-dan Chen, Fan Liao, Zhi-fei Wang

**Affiliations:** Department of Neurosurgery, The Third Xiangya Hospital, Central South University, 138 Tongzipo Road, Hexi Yuelu District, Changsha, Hunan 400013 China

**Keywords:** Glioma, miR-136, Sensitivity, Temozolomide, AEG-1

## Abstract

**Background:**

Recent studies have linked chemotherapy resistance to the altered expression of microRNAs (miRNAs). Thus, miRNA-based approaches to modulating sensitivity to temozolomide (TMZ) may overcome chemoresistance. The aim of the present study was to investigate whether miR-136 could modulates glioma cell sensitivity to TMZ.

**Methods:**

The proliferation of glioma U251 cell line was evaluated by MTT assay. The expression of astrocyte elevated gene-1 (AEG-1)was detected by real‑time polymerase chain reaction (RT-PCR)and Western blot. The luciferase reporter gene was used to test whether AEG-1 was the target of the miR-136.

**Results:**

The MTT assay showed that U251 cells with miR-136 overexpression were significantly more sensitive to the therapy of TMZ than control cells. Luciferase assays revealed that miR-136 directly targeted the 3’UTR of AEG-1. qRT–PCR and western blotting analysis found that AEG-1 expression at the mRNA and protein levels decreased in the miR-136 mimic-treatment group relative to control group. Downregulation of AEG-1 expression by siRNAs, U251 cells became more sensitive to the therapy of TMZ. In addition, the enhanced growth-inhibitory effect by the miR-136 mimics transfection was enhanced after the addition of AEG-1 siRNA.

**Conclusions:**

The present study provides the first evidence that miR-136 plays a key role in TMZ resistance by targeting the AEG-1 protein in glioma cell line, suggesting that miR-136 can be used to predict a patient’s response to TMZ therapy as well as serve as a novel potential maker for glioma therapy.

**Virtual Slides:**

The virtual slide(s) for this article can be found here: http://www.diagnosticpathology.diagnomx.eu/vs/13000_2014_173

## Background

Glioma is the most common type of primary brain tumor, originating in glial cells, with poor prognosis during the past 40 years [1]. Approximately 20,000 new cases of glioma are diagnosed in the United States every year[1]. Malignant gliomas, including glioblastoma multiforme (GBM) and anaplastic astrocytomas are the most common primary brain tumors [2]. GBM is a highly aggressive and neurologically destructive tumor that frequently colonizes the cerebral hemispheres. First-line postsurgical therapy for GBM currently consists of temozolomide (TMZ) combined with regional fractionated radiation followed by TMZ alone [3]. TMZ acts by inhibiting the proliferation of glioma cells and inducing apoptosis [4]. Although TMZ is currently the most promising chemotherapy for GBM, it is not always effective because of the intrinsic or acquired chemoresistance of glioma cells.

MicroRNAs (miRNAs) are a type of endogenous non-coding RNA, which are able to bind to the 3'-untranslated region (3'-UTR) of their target mRNAs, eventually causing mRNA degradation or translational repression [5]. In previous decades, accumulating studies have demonstrated that miRNAs act as key regulators in the development and progression of various types of cancer, including malignant glioma [6]. Recently, miRNA microarray expression profiling showed that miR-136 was overexpressed in lung cancer [7]. miR-136 was also reported to be markedly upregulated in the Jurkat cell line and targeted tumor suppressor PTEN, suggesting a possible significance of miR-136 in cancer development and progression [8,9]. Haapa-Paananen et al. found that the expression of miR-136 was lower in glioma tissues than that in normal brain tissues, suggesting that miR-136 might play a tumor-suppressive role in glioma [10]. Recent studies have linked chemotherapy resistance to the altered expression of microRNAs (miRNAs) [11,12]. Thus, miRNA-based approaches to modulating sensitivity to TMZ may overcome chemoresistance.

Astrocyte elevated gene-1 (AEG-1), was originally cloned as a novel human immunodeficiency virus (HIV)-1- and tumor necrosis factor-α-inducible gene in primary human fetal astrocytes (PHFA) [13]. Recent studies indicated that AEG-1 expression was elevated in some solid tumors including prostate, breast, esophageal cancer, hepatocellular carcinoma(HCC) and neuroblastoma [14-18]. The study by Emdad et al. indicated that AEG-1 might play a crucial role in the pathogenesis of glioma and that AEG-1 could represent a viable potential target for malignant glioma therapy [19].

We found that miR-136 might modulate AEG-1 using online prediction software Target Scan. Therefore, we speculated that miR-136 might play an important role in glioma chemoresistance by targeting AEG-1.

## Methods

### Cell culture and transfection

The human glioma cell line U251 was obtained from the American Type Culture Collection, and were cultured in Dulbecco’s modified Eagle’s medium (DMEM) supplemented with 10% fetal bovine serum (FBS), 100 units of penicillin/mL, and 100 ng of streptomycin/mL. Transfection of U251 cells with plasmids, pre-miR-136 or scrambled pre -miR control (GenePharma, Shanghai, China), was performed using Lipofectamine 2000 according to the manufacturer's instructions (Invitrogen Life Technologies, Carlsbad, CA, USA). Untransfected U251 cells were employed as a negative control.

Total RNA extraction and real‑time polymerase chain reaction (qRT-PCR). Total RNA was extracted from cells using a modified TRIzol one‑step extraction method (Invitrogen Life Technologies). Stem-loop reverse transcription for mature miR-136 and U6 primers was performed. U6 RNA was used as an miRNA internal control. The primers used for stem-loop reverse -transcription PCR for miR-136 were purchased from Guangzhou RiboBio Co., Ltd. (Guangzhou, China). Each sample was conducted in triplicate and the results were calculated using the 2^-ΔΔCt^ method.

### MTT assay

Cells transfected with plasmids, pre-miR-136 or scrambled pre-miR control or AEG-1 siRNA, were seeded into 96-well plates at 6*10^3^ cells/well and allowed to grow overnight, and then were treated with different concentrations of TMZ. After 24 h of treatment, 20ul of 5 mg/ml MTT reagent (Sigma-Aldrich,St.Louis, MO, USA) was added and incubated in the dark for 4 h. The absorbance of the plate was measured in a microplate reader at a wavelength of a 570-nm reference (BMG Lab Technologies, Germany), and the results were expressed as the percentage of absorbance relative to untreated controls. Each treatment was carried out in triplicate.

### Dual luciferase reporter assay

The construction of psiCHECK- AEG-1 and psiCHECK-AEG-1^mut^ 3’ UTRs was performed and verified by DNA sequencing. A total of 293 T cells (5 × 10^4^ per well) were seeded into 24-well plates and allowed to attach overnight. The cells were cotransfected with a 50 nM miR-136 mimic or control, as well as a 0.5ug reporter vector containing either MEK1 3’UTRs or MEK1^mut^ 3’ UTRs carrying a mutational miR-136 binding site. After 48 h, cells were harvested and lysed, and ratios between renilla and firefly luciferase activity were estimated using the Dual Luciferase Assay Kit (Promega) according to the manufacturer’s instructions. Data were presented as the mean value of renilla/firefly luciferase ratios, obtained from at least three independent experiments.

### Western blot assay

The proteins were resolved on an SDS denaturing polyacrylamide gel and then transferred onto a nitrocellulose membrane. Antibody to AEG-1 or GAPDH was incubated with the membranes overnight at 4°C. The membranes were washed and incubated with horseradish peroxidase (HRP)-conjugated secondary antibodies. Protein expression was assessed by enhanced chemiluminescence and exposure to chemiluminescent film. LabWorks™ Image Acquisition and Analysis Software (UVP, Upland, CA) were used to quantify the band intensities. All the antibodies were purchased from Abcam (Cambridge, MA).

### Statistical analysis

All the data were shown as mean ± standard deviation (SD) and the experiments.

Were repeated three times. The difference was determined by two-tailed students’ t-test and *P* < 0.05 was considered statistically significant. The data were assessed using the GraphPad Prism software 5.0 (GraphPad, USA) and SPSS version 18.0 (SPSS,Chicago, IL, USA).

## Results

### Overexpression of miR-136 correlates with cytotoxic activity of TMZ in U251 cells

**Figure 1:**
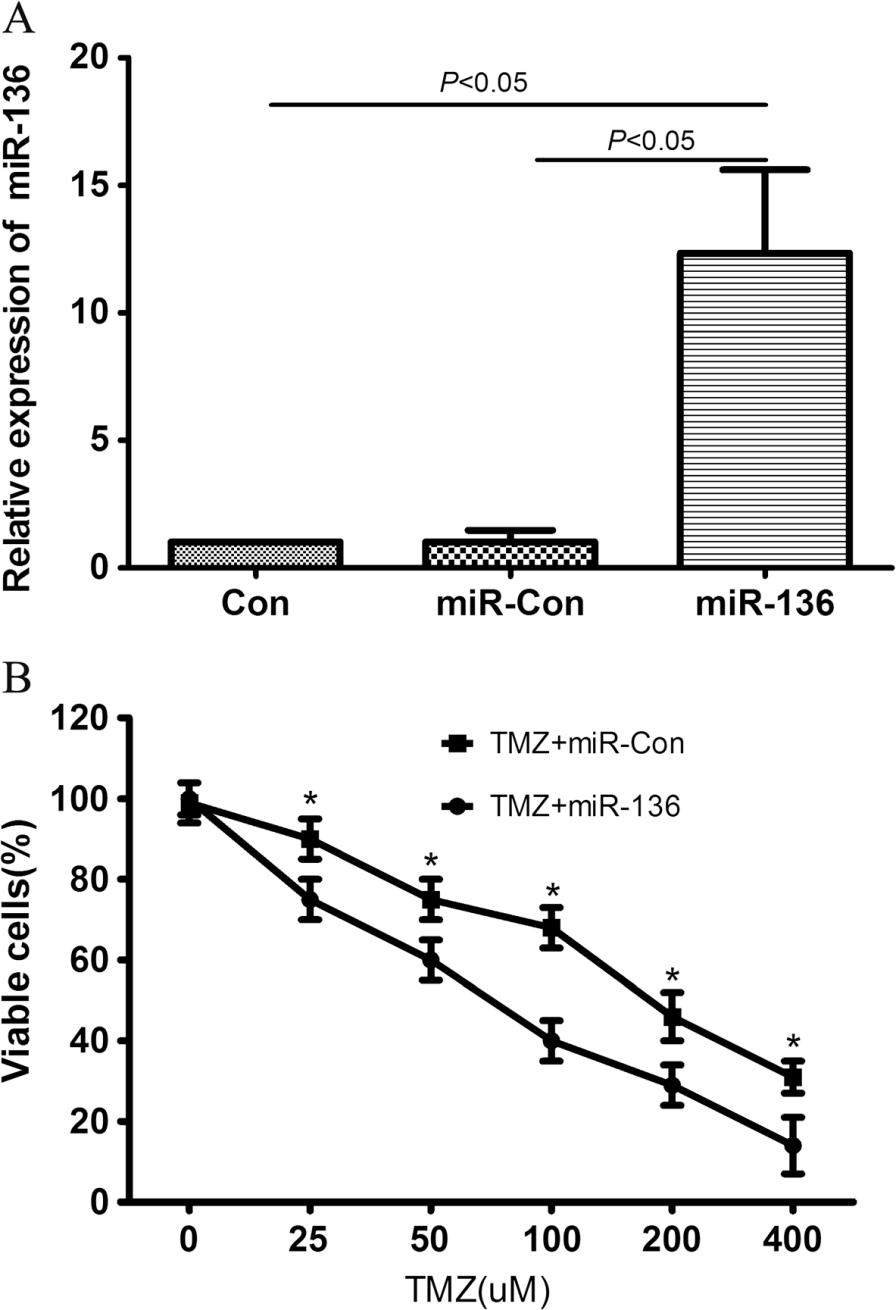
**Effect of the overexpression of miR‑136 on the cytotoxic activity of TMZ in U251 cells. (A)** miR‑136‑transfected cells showed higher miR‑136 expression than the Con (*P* < 0.05) and miR-Con (*P* < 0.05) groups. **(B)** Cell viability was detected by MTT assay in pre-miR-136 transfected cells and Con and miR-Con groups following treatment with TMZ for 24 h.*P < 0.05.

In order to explore the role of miR-136 in U251 cells, transfection with plasmids, pre-miR-136 or scrambled pre-miR control, was performed. The expression Levels of miR −136 were confirmed by qPCR, as shown in Figure 1A. Pre- miR-136 (miR −136) - transfected cells showed a higher miR-136 expression than the untransfected negative control (Con) and empty vector-transfected (miR-Con) groups. To evaluate the effect of miR-136 on the cytotoxic activity of TMZ in U251 cells, MTT assay was performed on the pre- miR-136 transfected cells, the Con and miR-Con groups combined with various concentrations of TMZ. The results showed that the viability of U251 cells with miR-136 overexpression was significantly decreased compared with that of the miR-Con group at the same concentration of

TMZ (shown in Figure 1B). This indicated that miR‑136 sensitized U251 cells to TMZ compared with the Con group. This observation suggested that the overexpression of miR-136 facilitated the cytotoxic activity of TMZ in U251 cells.

### AEG-1 was a target of miR-136

**Figure 2:**
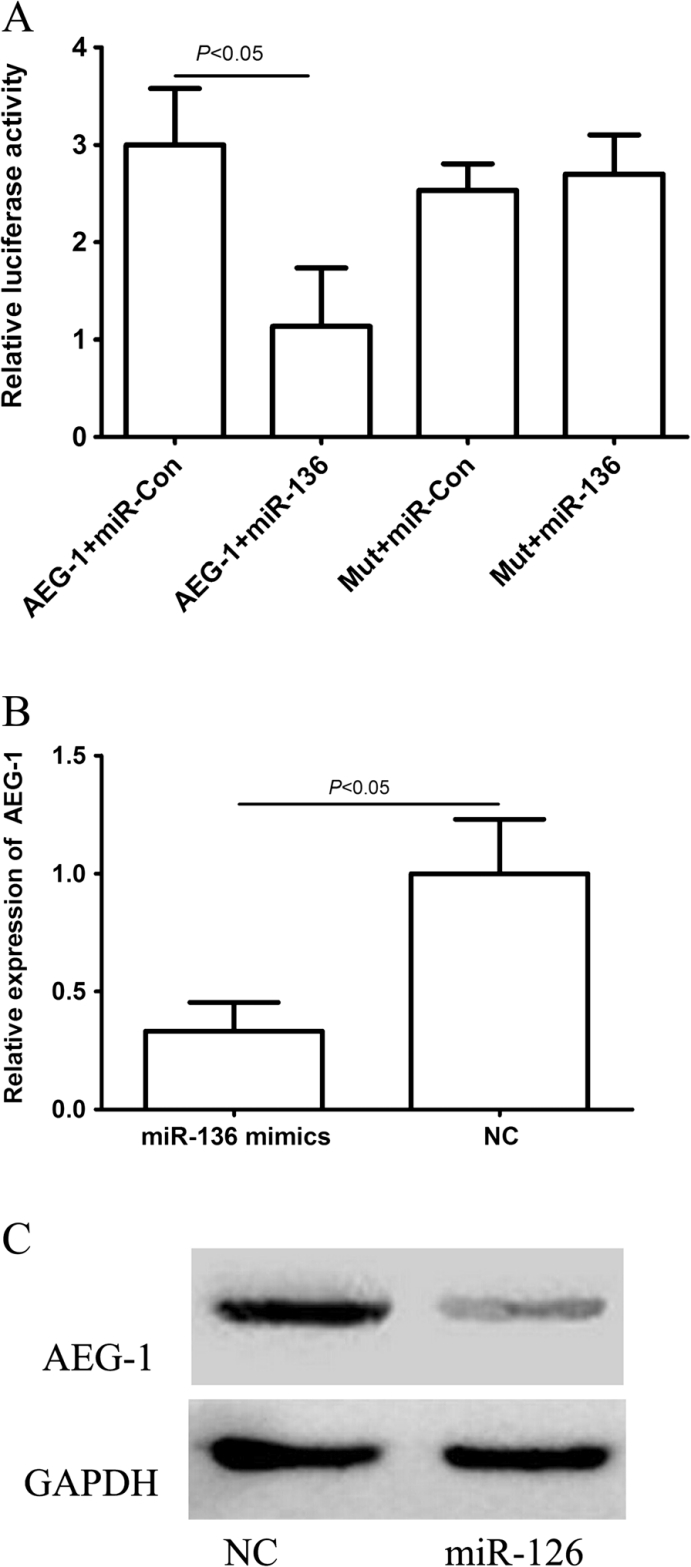
**AEG-1 was a direct target of miR-136. A**: the luciferase activity significantly decreased after co-transfection with psiCHECK-2/AEG-1 3’-UTR and miR-136 mimics in comparison with control cells. **B**: AEG-1 mRNA level was detected by qRT–PCR in U251 cells transfected with miR-136 mimic or the control. **C**: AEG-1 protein level was detected by Western blotting in U251 cells transfected with miR-136 mimic or the control.

Predicted miR-136 targets were identified using the algorithms of TargetScan and microRNA. Among hundreds of potential target genes, we specifically focused on AEG-1, as it has been consistently reported to be oncogene in various cancers. We identified a binding site for miR-136 in the 3’-UTR of AEG-1 mRNA. To confirm that miR-136 can bind to the predicted site, we performed a luciferase reporter assay in the 293 T cell line. Figure 2A showed that the luciferase activity significantly decreased after co-transfection with psiCHECK-2/ AEG-1 3’-UTR and miR-136 mimics in comparison with control cells. The results demonstrated that miR-136 specifically binded to the 3’-UTR of AEG-1 mRNA. The effect of miR-136 transfection on AEG-1 mRNA and protein expression was respectively assessed using qRT-PCR and Western blot in U251 cell line. As shown in Figure 2B and C, the qRT–PCR and western blotting analysis found that AEG-1 expression at the mRNA and protein levels decreased in the miR-136 mimic-treatment group relative to NC.

### AEG-1 was responsible for the miR-136-induced resistance to TMZ in U251 cells

**Figure 3:**
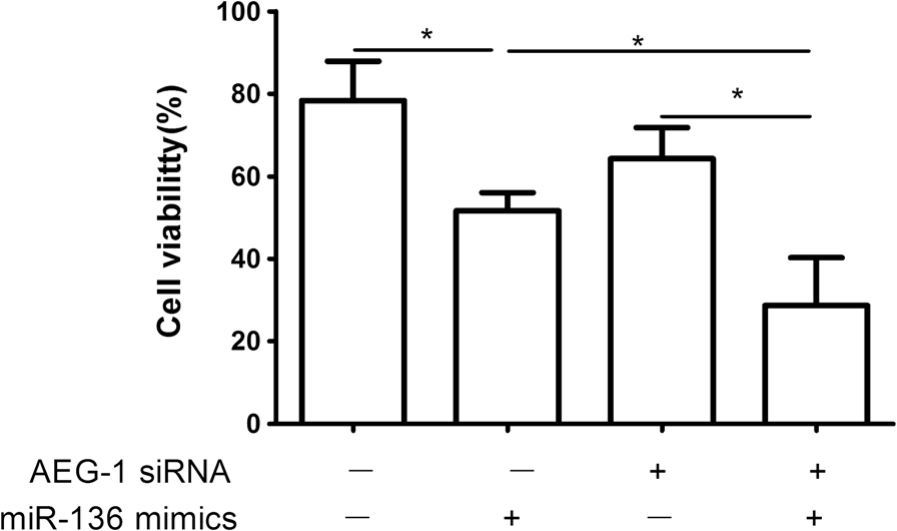
**Downregulation of AEG-1 expression by siRNAs, U251 cells became more sensitive to the therapy of TMZ.** In addition, the enhanced growth-inhibitory effect by the miR-136 mimics transfection was enhanced after the addition of AEG-1 siRNA. *P < 0.05.

Downregulation of AEG-1 expression by siRNAs, U251 cells became more sensitive to the therapy of TMZ (shown in Figure 3). In addition, the enhanced growth-inhibitory effect by the miR-136 mimics transfection was enhanced after the addition of AEG-1 siRNA(shown in Figure 3).

## Discussion

TMZ, which inhibits glioma cell growth and induces apoptosis, has been adopted as the first-line drug in patients with glioma [20]. However, it has produced only modest improvements in disease outcomes, attributed in part to the presence of existing or de novo resistance to chemotherapy in glioma patients. Therefore, it is worth-while to select the correct anticancer drug to the right cancer patient. Predictive markers, predicting a response, often related to individually tailored therapy.

MicroRNAs, a class of small regulatory RNAs, have been demonstrated to play important roles in a wide variety of oncogenic activities, such as proliferation, invasion, and angiogenesis [21]. Dysregulated miRNAs have been observed in various kinds of tumors, including brain tumors such as glioma and its aggressive glioblastoma subtype [22]. Accumulating data indicate that miRNAs are involved in advanced stages of cancer progression and may act as activators or suppressors of tumorigenesis. Recent studies have strongly implicated aberrant miRNA expression in anticancer drug resistance and sensitivity [12,23,24]. In glioma, Shi et al. found that miR-124 inhibited glioma cell growth, invasion, angiogenesis, and tumor growth and increased chemosensitivity to TMZ treatment by negatively regulating the Ras family and its downstream signaling pathways: phosphatidylinositol-3 kinase/Akt and Raf/extracellular signal-regulated kinase 1/2 [25]. Wang et al. found that glioma cells rich in miR-181b were more sensitive to TMZ therapy. miR-181b combined with TMZ enhanced glioma cell sensitivity and apoptosis. The effects were through post-transcriptional repression of MEK1. Therefore, a combination of miR-181b and TMZ might be an effective therapeutic strategy for gliomas [26]. Recently, miRNA microarray expression profiling showed that miR-136 was overexpressed in lung cancer [7]. miR-136 was also reported to be markedly upregulated in the Jurkat cell line and targeted tumor suppressor PTEN, suggesting a possible significance of miR-136 in cancer development and progression [8,9]. Latter reports showed that miR-136 was implicated in cancer biology and played roles as both tumor suppressor and oncogene in different types of cancer by targeting distinct genes. Haapa-Paananen et al. found that the expression of miR-136 was lower in glioma tissues than that in normal brain tissues, suggesting that miR-136 might play a tumor-suppressive role in glioma [10]. In the present study, to evaluate the effect of miR-136 on the cytotoxic activity of TMZ in U251 cells, MTT assay was performed on the pre- miR-136 transfected cells, the Con and miR-Con groups combined with various concentrations of TMZ. The results showed that the viability of U251 cells with miR-136 overexpression was significantly decreased compared with that of the miR-Con group at the same concentration of TMZ, indicating that miR‑136 sensitizes U251 cells to TMZ compared with the Con group. This observation suggested that the overexpression of miR-136 facilitates the cytotoxic activity of TMZ in U251 cells. As we know, many onco-miRs/tumor suppressor-target or tumor suppressor-miRs/onco-target pathways have been demonstrated to participate in the tumorigenesis of glioma. However, miRNA/target network was so complex that more and more miRNA/target axis needs to be elucidated in glioma. Predicted miR-136 targets were identified using the algorithms of TargetScan and microRNA. Among hundreds of potential target genes, we specifically focused on AEG-1, as it has been consistently reported to be oncogene in various cancers. Recent studies indicate that AEG-1 expression is elevated in some solid tumors including prostate, breast, esophageal cancer, HCC and neuroblastoma [14-18]. The study by Emdad et al. indicated that AEG-1 might play a crucial role in the pathogenesis of glioma and that AEG-1 could represent a viable potential target for malignant glioma therapy[19]. In the present study, luciferase assays revealed that miR-136 directly targeted the 3’UTR of AEG-1. In addition, qRT–PCR and western blotting analysis found that AEG-1 expression at the mRNA and protein levels decreased in the miR-136 mimic-treatment group relative to control group. Furthermore, downregulation of AEG-1 expression by siRNAs, U251 cells became more sensitive to the therapy of TMZ. In addition, the enhanced growth-inhibitory effect by the miR-136 mimics transfection was enhanced after the addition of AEG-1 siRNA. These findings suggested that AEG-1 was responsible for the miR-136-induced resistance to TMZ. Our study might provide a useful strategy to overcome TMZ resistance in glioma. The present findings are needed to be further validated by in vivo experiments and clinical studies. In addition, elucidation of role of other miRNAs in TMZ resistance requires further study.

## Conclusion

In conclusions, the present study provides the first evidence that miR-136 plays a key role in TMZ resistance by targeting the AEG-1 protein in glioma cell line, suggesting that miR-136 can be used to predict a patient’s response to TMZ therapy as well as serve as a novel potential maker for glioma therapy.
